# Distinct systemic metabolic signatures in premenopausal women with lipedema revealed by composite indices

**DOI:** 10.3389/fendo.2026.1857893

**Published:** 2026-06-05

**Authors:** Sally Kempa, Christa Buechler, Lukas Prantl, Martina Müller, Christian Sina, Franziska Schmelter, Claudia Kunst, Karsten Gülow, Kristina Büscher, Lorena Rudolph, Ulrich L. Günther, Jens U. Marquardt, Hauke C. Tews, Melanie Kandulski, Lina Jegodzinski

**Affiliations:** 1 Department of Plastic, Hand, and Reconstructive Surgery, University Hospital Regensburg, Regensburg, Germany; 2 Department of Internal Medicine I, Gastroenterology, Hepatology, Endocrinology, Rheumatology, and Infectious Diseases, University Hospital Regensburg, Regensburg, Germany; 3 Institute of Nutritional Medicine, University of Lübeck, Lübeck, Germany; 4 Department of Medicine I, University Hospital Schleswig-Holstein, University of Lübeck, Lübeck, Germany; 5 College of Medicine and Health Sciences, Khalifa University, Abu Dhabi, United Arab Emirates; 6 Institute of Chemistry and Metabolomics, University of Lübeck, Lübeck, Germany

**Keywords:** composite indices, fat distribution, lipedema, metabolism, metabolomics

## Abstract

**Background:**

Lipedema is a chronic adipose tissue disorder with disproportionate fat accumulation in the extremities and is often misdiagnosed as obesity. Although women with lipedema appear to be metabolically distinct from body mass index (BMI)-matched controls, their fasting metabolism remains insufficiently characterized. We therefore aimed to define the metabolic signature of lipedema using serum NMR metabolomics and anthropometric profiling.

**Methods:**

We conducted a study with 24 premenopausal women with lipedema and 21 BMI-matched controls. Fasting serum samples were analyzed using NMR spectroscopy and anthropometric data were collected. Regional body composition was additionally assessed in an exploratory matched DXA subset (n=12). To characterize coordinated metabolic differences beyond single analytes, we derived exploratory composite indices and applied multivariate analyses.

**Results:**

Despite similar BMI, women with lipedema showed lower waist circumference, waist-to-hip ratio and lower fasting insulin than controls (age-adjusted *p* = 0.032). NMR profiling revealed lower alanine (*p* < 0.001), lactate (*p* = 0.004), pyruvate (*p* = 0.021), and elevated ketone bodies (3-hydroxybutyric acid: *p* = 0.009; acetoacetic acid: *p* = 0.035; acetone: *p* = 0.006). These alterations were reflected by significant group differences in composite indices for fat distribution (*g* = 1.26; *p* < 0.001), glycolysis (*g* = 0.74; *p* = 0.018), and ketone metabolism (*g* = 0.70; *p* = 0.018). Principal component analysis of the selected indices explained 78% of the total variance and showed partial group separation between lipedema and controls.

**Conclusion:**

Lipedema is associated with a distinct fasting metabolic profile characterized by reduced glycolytic intermediates, enhanced ketone body signals, and a more peripheral fat distribution despite comparable BMI. These findings support the concept of lipedema as a metabolically distinct phenotype and suggest that multivariate metabolic signatures may help refine future diagnostic and interventional approaches.

## 1 Introduction

Recently, the Lipedema World Alliance Delphi Consensus (1) established a new global standard for the definition of lipedema, focusing on several core clinical features. Firstly, lipedema usually presents as a symmetrical, bilateral enlargement of subcutaneous adipose tissue in the extremities, which is disproportionate compared to the torso, sparing the hands and feet, and with a negative Kaposi-Stemmer sign (1). Secondly, common symptoms include a persistent sensation of swelling or heaviness, increased sensitivity to pressure or stretching (often described by patients as pain), and are restricted to areas with lipedema-related volume increase (1). Finally, unlike lymphedema, pitting edema is usually absent in the affected tissue, and patients frequently experience easy bruising in these areas (1). The onset or progression of symptoms often coincides with hormonal changes such as puberty, pregnancy, or menopause, suggesting an underlying endocrine component (2). Despite increasing awareness, lipedema remains underdiagnosed and insufficiently understood. Diagnosis currently relies on clinical examination and history, as there are no standardized diagnostic tests, such as blood biomarkers or imaging tools, available for confirmation (3).

Beyond the clinical phenotype, lipedema is increasingly recognized as a complex condition defined by a unique interplay between local tissue pathology and systemic metabolic adaptations (4). At the systemic level, glucose metabolism reflects the “metabolic paradox” of lipedema. We use the term to describe the observation that women with lipedema often present with a high body mass index (BMI) due to disproportionate subcutaneous fat accumulation, yet show comparatively preserved insulin sensitivity (5, 6) and a lower prevalence of type 2 diabetes than expected for their BMI (7, 8).

By effectively storing lipids in the extremities, the tissue apparently shields the liver and visceral organs from ectopic fat accumulation and lipotoxicity (5, 9). In contrast, the systemic lipid profile presents a heterogeneous and often inconsistent picture. While some BMI-matched studies report a more favorable profile with lower LDL-cholesterol (low-density lipoprotein cholesterol) and triglycerides (6, 10), others have observed higher cholesterol levels in lipedema (11, 12). This heterogeneity likely reflects differences in disease stages (7) or modest sample sizes and suggests that many lipid alterations observed in lipedema may primarily reflect the underlying degree of obesity rather than lipedema-specific pathology (13). Furthermore, the characteristic resistance to traditional weight-loss treatments of pathological lipedema tissue suggests a disconnection between systemic lipid levels and local lipid turnover.

Since the previous focus on isolated individual parameters was unable to fully characterize this complex phenotype, we conducted comprehensive metabolic profiling in the fasting state in premenopausal women with lipedema and BMI-matched female controls. We hypothesized that lipedema is best captured by a multivariate metabolic signature rather than by single-analyte differences. We therefore derived eight exploratory composite scores as interpretable summaries of coordinated metabolite patterns and applied multivariate analyses to assess group separation, providing interpretable readouts for future longitudinal and interventional studies.

## 2 Methods

### 2.1 Study design and participants

This is a cross-sectional observational study involving 24 premenopausal women with clinically confirmed lipedema and 21 premenopausal female controls with comparable BMI and no clinical signs of lipedema. Lipedema diagnosis was established during routine clinical assessment at the University Hospital of Regensburg by experienced clinicians in accordance with standardized clinical criteria consistent with the Lipedema World Alliance Delphi Consensus (1). Participants with lipedema had no clinical signs of lymphedema (negative Stemmer sign and absence of pitting edema) and exhibited the typical lipedema phenotype with symmetrical extremity fat accumulation, sparing of the feet and associated symptoms such as heaviness and easy bruising. Exclusion criteria included pregnancy, breastfeeding, malignant diseases, active infection, severe or untreated organ diseases such as heart, kidney, liver, thyroid, or lung conditions, surgical procedures for weight reduction, liposuction, use of weight-loss medication, positive tests for HIV, hepatitis B, and hepatitis C, chronic use of systemic glucocorticoids, atypical antipsychotic medications, or insulin intake. It should be noted that neither the use of hormonal contraception nor the diagnosis of metformin-treated type 2 diabetes was an exclusion criterion. While hormonal contraception was used by participants in both groups, metformin-treated diabetes was present only in the control group, where four individuals were being treated with metformin. Metformin-treated type 2 diabetes was not an exclusion criterion to avoid selecting a metabolically healthy control group within the obese BMI range.

**Figure 1:**
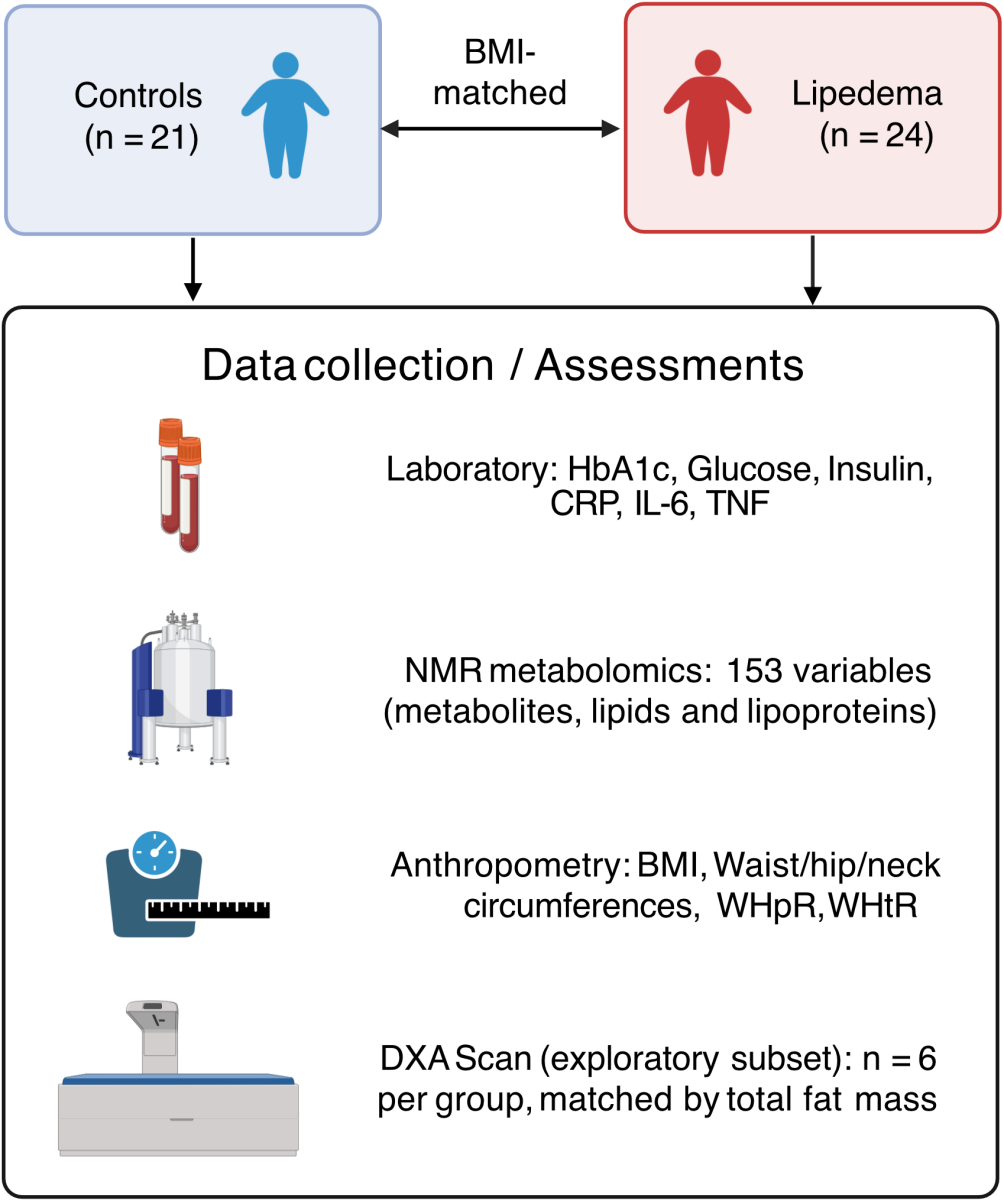
Study design and workflow (created with BioRender.com). This diagram illustrates the cross-sectional study design involving 24 premenopausal women with lipedema and 21 BMI-matched female controls. Data collection included laboratory analyses (HbA1c, glucose, insulin, CRP, IL-6, TNF), NMR metabolomics (153 variables), and anthropometric measurements (BMI, waist, hip, and neck circumferences, waist-to-hip ratio (WHpR), waist-to-height ratio (WHtR)). An exploratory subset (n=6 per group), matched for total fat mass, underwent DXA scanning.

The study was conducted in accordance with the Declaration of Helsinki and approved by the responsible ethics committee (reference number 22-3163-101, date of approval: 15.12.2022; University Hospital of Regensburg). Patient recruitment and data collection took place between March 2024 and March 2025. Participants were recruited consecutively during routine clinical care or through local networks. All participants provided written informed consent after receiving detailed information. The sample size was primarily determined by the feasibility and availability of clinically confirmed lipedema patients who met all the inclusion criteria within the recruitment period. The overall study design and workflow are outlined in **Figure 1**.

In addition, a previously published exploratory pilot cohort (“pre-study”) consisting of non-fasting serum samples from lipedema patients (13) was included in a secondary analysis. While not directly comparable due to differences in fasting status, these data were used to externally project the pre-study samples into the principal component space derived from the main study cohort, providing an independent reference for interpretability.

### 2.2 Baseline data collection

Baseline assessments were performed at the study visit. After an overnight fast of at least 8 hours, venous blood samples were collected for routine laboratory testing. Anthropometric measurements included weight, height, body mass index (BMI), waist circumference, hip circumference, neck circumference, waist-to-hip ratio (WHpR), and waist-to-height ratio (WHtR). Blood pressure was measured on the dominant arm after a 5-minute seated rest. Routine laboratory parameters included HbA1c, fasting glucose, fasting insulin, triglycerides, HDL cholesterol, and C-reactive protein (CRP). Inflammatory cytokines interleukin-6 (IL-6) and tumor necrosis factor (TNF) were measured using standard methods. Physical activity was assessed as the mean daily step count over the preceding 7 days. Special dietary patterns (e.g., intermittent fasting, ketogenic diet, or other restrictive regimens) were recorded by self-report at baseline and reported descriptively. Although all participants confirmed fasting for at least 8 hours before blood sampling, exact fasting duration, quantitative macronutrient intake, and the duration of specific dietary regimens were not recorded.

### 2.3 NMR metabolomics

In addition, a comprehensive metabolomic analysis was performed using nuclear magnetic resonance spectroscopy (NMR), which quantified various metabolites such as amino acids, ketone bodies, glycolysis markers, as well as lipoproteins (particles) (a total of 153 variables). Samples were prepared following a previously described standard operating procedure (SOP) (13, 14). NMR spectra were acquired within a maximum of 24 h after sample preparation. All NMR experiments were performed on a Bruker Avance III HD 600 MHz NMR spectrometer equipped with a TXI room temperature probe and a Bruker SampleJet™ automatic sample exchanger with sample storage set at 6°C. All experiments were conducted at 310K. Temperature precision, quantification accuracy, water suppression performance, and gradient profiles were tested and calibrated daily following Bruker’s SOPs. For the metabolite quantification experiments, 32 scans were acquired with a relaxation delay (D1) of 4 s. For lipoprotein subclass analysis, the mixing time (D8) was set to 0.01 s. Samples were analyzed in the order received and were therefore not fully randomized. However, lipedema and control samples were not processed as two separate group-wise blocks. All measurements were performed on the same NMR platform under full automation, with daily calibration and quality-control procedures according to Bruker SOPs, thereby minimizing potential batch-related variability. Bruker Quantification in Plasma/Serum (B.I.Quant-PS 2.0.0) and Bruker IVDr Lipoprotein Subclass Analysis (B.I.-LISA, Bruker Corporation (Billerica, MA, USA)) were used to automatically quantify 39 metabolites (+2 technical additives) and 112 lipoprotein parameters, including very-low density lipoprotein (VLDL), intermediate-density lipoprotein (IDL), low-density lipoprotein (LDL), and high-density lipoprotein (HDL). All of these major classes were further categorized into their subfractions, which were characterized by their content of triglycerides (TGs), cholesterol (CH), free cholesterol (FC), phospholipids (PL), and apolipoproteins (Apo).

### 2.4 Body composition assessment by dual energy X-ray absorptiometry

Body composition was assessed using dual energy X-ray absorptiometry (DXA, Hologic, Apex software). Due to the limited availability of DXA scans for the entire cohort, a small, explorative subset (n = 12) of 6 controls and 6 matched lipedema patients was selected for this specific analysis. To control for potential confounding effects of overall fat mass, the two groups were matched 1:1 using Propensity Score Matching based on total fat mass. The DXA scan provided detailed body composition data, including: 1. fat mass (arms, trunk, legs, head, and total); 2. android and gynoid fat mass; 3. total body fat distribution; 4. body fat mass/height²; ratios such as android-gynoid ratio, trunk fat/leg fat, and adjusted indices for fat distribution, including leg fat-BMI-adjusted, leg-trunk BMI-adjusted, and android-gynoid BMI-adjusted.

### 2.5 Composite metabolic and body composition indices

To characterize group differences beyond individual analytes, we newly derived eight composite indices (**Table 1**). The calculation followed a standardized three-step process to ensure comparability and biological interpretability. First, all constituent variables were z-standardized (mean = 0, standard deviation = 1) based on the total study population to normalize markers with different units of measurement (15). Second, we applied a directional alignment (orientation) to each component. For variables that were decreased in the patients with lipedema group (e.g., alanine, lactate, or pyruvate), the z-scores were multiplied by -1 to facilitate graphic presentation. Finally, the composite index was calculated as the row-wise mean of these oriented z-scores. The lipoprotein index was defined as the difference between the mean z-score of the “atherogenic” set and the “anti−atherogenic” set.

**Table 1:** Composition and interpretation of the composite indices.

Index	Domain	Variables	Interpretation of higher values
1. Fat distribution	Body composition	WHpR, WHtR, trunk/leg fat ratio, trunk/extremities fat ratio, android/gynoid ratio (BMI-adjusted)	Higher peripheral/gynoid fat distribution (lipedema-typical)
2. Fat amount		BMI, total fat mass, body fat mass/height²	Higher overall degree of systemic obesity
3. Glycolysis	Energy metabolism	Lactate, pyruvate	Lower systemic glycolytic activity
4. Ketone bodies		3-Hydroxybutyrate, acetoacetic acid	Higher levels of circulating ketone bodies
5. Triglycerides/glycerol		Triglycerides, glycerol	Higher lipid mobilization or basal turnover
6. Branched-chain amino acids	Amino acids	Valine, leucine, isoleucine	Altered branched-chain amino acid profile
7. Aromatic amino acids		Tyrosine, phenylalanine	Altered aromatic amino acid profile
8. Lipoprotein*	Cardiovascular risk	LDL/HDL, ApoB100/ApoA1 and VLDL particles minus HDL	More atherogenic lipoprotein profile

WHpR, waist-to-hip ratio; WHtR, waist-to-height ratio.

*Lipoprotein index: Defined as mean z-score of atherogenic set (LDL/HDL ratio, ApoB100/ApoA1 ratio, VLDL particle count), minus the mean z-score of HDL (anti-atherogenic).

### 2.6 Statistical analyses

All statistical analyses were performed in R (version 4.5.1; R Core Team, 2025) using RStudio (version 2025.05.1; Posit Software, Boston, MA, USA). Continuous variables were tested for normality and compared between groups using either t-tests (with Welch’s correction if appropriate) or Mann–Whitney U-tests. To account for the age difference between groups, primary comparisons were repeated in an ANCOVA (Analysis of Covariance) framework, adjusting for age (**Supplementary Table 1, S2, S6**). False discovery rate (FDR) was controlled using the Benjamini–Hochberg method, with *q* < 0.05 considered significant. Effect sizes are reported as Hedges’ *g* with 95% confidence intervals. Correlations between metabolic and anthropometric parameters were assessed using Spearman’s rank correlation coefficient. Composite metabolic indices were calculated and used for group comparisons and principal component analysis (PCA). Sensitivity analyses were performed to assess robustness to potential confounding by medically managed type 2 diabetes in the control group by repeating key analyses after excluding these participants. In addition, selected models were further adjusted for fasting insulin and age. Full details are provided in the **Supplementary Methods**.

## 3 Results

### 3.1 Baseline characteristics

The baseline characteristics of the study population are summarized in **Table 2**. The average age of the lipedema patients was significantly higher than that of the control group, with a mean of 41.0 ± 6.0 years compared to 35.8 ± 7.6 years (*p* = 0.013). Therefore, all subsequent statistical comparisons between groups were performed using ANCOVA (analysis of covariance) models adjusted for age, unless stated otherwise. The BMI was comparable between the two groups, with values of 36.8 ± 8.0 kg/m² for the lipedema patients and 33.5 ± 6.6 kg/m² for the controls (p = 0.141).

**Table 2:** Baseline characteristics of the two groups (controls and patients with lipedema).

Variable	Control (n=21)	Lipedema (n=24)	SMD	*P*-value	*P* adj (age)
Age (years)	35.8 ± 7.6	41.0 ± 6.0	0.77	**0.013**	NA
BMI (kg/m²)	33.5 ± 6.6	36.8 ± 8.0	0.45	0.141	0.127
Physical activity (steps/day)*	8000 [7000–9000]	7695 [6314–9598]	0.022	0.80	0.84
Diet: no special diet, n (%)	20 (95)	20 (84)	–	–	–
Diet: vegetarian, n (%)	0 (0)	1 (4)	–	–	–
Diet: intermittent fasting, n (%)	0 (0)	1 (4)	–	–	–
Diet: ketogenic/low-carbohydrate, n (%)	0 (0)	0 (0)	–	–	–
Diet: not reported, n (%)	1 (5)	2 (8)	–	–	–
Neck circumference (cm)*	40.0 [40.0-40.8]	36.8 [34.2-38.0]	-1.12	**0.003**	**0.012**
Waist circumference (cm)	106.6 ± 15.9	95.8 ± 13.0	-0.75	**0.020**	**0.032**
Hip circumference (cm)	123.8 ± 12.1	127.5 ± 13.2	0.29	0.352	0.353
Waist-to-hip ratio (WHpR)	0.86 ± 0.08	0.75 ± 0.08	-1.4	**<0.001**	**<0.001**
Waist-to-height ratio	0.64 ± 0.09	0.57 ± 0.08	-0.72	**0.024**	**0.030**
Systolic blood pressure (mmHg)	127.0 [115.0-145.0]	128.0 [120.0-146.0]	0.21	0.655	0.768
Diastolic blood pressure (mmHg)	80.0 [77.0-86.0]	83.0 [75.0-87.5]	0.09	0.934	0.997
Fasting glucose (mmol/L)	4.9 ± 0.8	4.6 ± 0.5	-0.35	0.262	0.071
Fasting insulin (µU/mL)*	14.6 [9.3-22.6]	5.8 [4.1-11.2]	-0.77	**0.015**	**0.032**
HOMA-IR*	2.9 [1.9-5.3]	1.1 [0.8-2.6]	-0.8	**0.017**	**0.031**
HbA1c (%)	5.5 [5.3-5.6]	5.5 [5.4-5.8]	0.13	0.521	0.790
Type 2 diabetes (n, %)	4 (19)	0 (0)	–	–	–
Metformin use (n, %)	4 (19)	0 (0)	–	–	–
Triglycerides (mg/dL)	116.1 [62.5-134.1]	89.6 [70.6-113.7]	-0.53	0.270	0.258
HDL cholesterol (mg/dL)	51.9 ± 9.1	54.1 ± 10.2	0.23	0.444	0.406
CRP (mg/L)	3.1 [1.4–6.7]	3.6 [1.3–9.8]	-0.21	0.814	0.158
IL-6 (pg/mL)*	4.0 [3.4–4.3]	4.2 [3.1–5.4]	-0.4	0.597	0.229
TNF (pg/mL)*	10.9 [10.1–11.0]	11.1 [9.2–13.2]	-0.36	0.342	0.395

*Indicates missing values >10%. The sample sizes for physical activity (C: n=15, L: n=20), neck circumference (C; n=14), fasting insulin (C; n=11, L; n=14), HOMA-IR (C; n=11, L; n=14), IL-6 (C; n=10, L; n=14) and TNF (C; n=9) are lower due to the availability of measurements.

Values are mean ± SD or median [IQR], depending on normality (Shapiro-Wilk). Group comparison by t test or Mann-Whitney U as appropriate. Age adjusted *p* values from ANCOVA, not computed for age. Effect sizes are standardized mean differences (SMD; Lipedema - Control). Bold values indicate statistically significant results.

Despite similar BMI values, patients with lipedema exhibited a significantly lower waist-to-hip ratio (0.8 ± 0.1 *vs*. 0.9 ± 0.1, *p* < 0.001) and smaller neck and waist circumferences, characteristics of the lipedema phenotype. Specifically, neck circumference was 36.8 cm (range: 34.2-38.0 cm) for lipedema patients compared to 40.0 cm (range: 40.0-40.8 cm) for controls (*p* = 0.012), while waist circumference measured 95.8 ± 13.0 cm for lipedema patients and 106.6 ± 15.9 cm for the control group (*p* = 0.032). There were no significant differences in hip circumference between the groups. These anthropometric differences remained significant even after adjusting for age. Physical activity, assessed as the self-reported mean daily step count over the preceding 7 days, did not differ between groups (**Table 3**). All participants reported an overnight fast of at least 8 hours, although the exact fasting duration was not quantitatively recorded. Regarding habitual diet, the majority of participants in both groups reported no specific dietary regimen (20/22 women with lipedema with available data and 20/21 controls). Dietary information was unavailable for 2 women with lipedema and 1 control. Two women with lipedema reported a restrictive pattern (one vegetarian, one practicing intermittent fasting). Notably, no participant in either group reported a ketogenic, low-carbohydrate, or other carbohydrate-restricted diet. Detailed quantitative dietary intake data, including macronutrient composition and the duration of dietary patterns, were not collected.

**Table 3:** Univariate comparisons of selected metabolic parameters across study groups.

Variable	Controls (median (range)	Lipedema (median (range)	Effect (log2)	95% CI	*P*-value (age-adj.)	FDR q-value
Alanine	0.40 [0.34–0.44]	0.32 [0.26–0.41]	-0.085	[-0.133, -0.038]	**<0.001**	**0.021**
Lactic acid	2.30 [1.80–2.40]	1.60 [1.48–1.95]	-0.844	[-1.400, -0.287]	**0.004**	0.086
Acetone	0.02 [0.01–0.03]	0.03 [0.02–0.06]	0.028	[0.008, 0.047]	**0.006**	0.093
3-Hydroxy-butyric acid	0.00 [0.00–0.05]	0.04 [0.02–0.16]	0.120	[0.032, 0.207]	**0.009**	0.114
Pyruvic acid	0.11 [0.09–0.14]	0.09 [0.08–0.11]	-0.023	[-0.042, -0.004]	**0.021**	0.178
Isoleucine	0.06 [0.05–0.06]	0.05 [0.04–0.05]	-0.012	[-0.022, -0.001]	**0.034**	0.217
Acetoacetic acid	0.00 [0.00–0.01]	0.01 [0.00–0.03]	0.024	[0.002, 0.046]	**0.035**	0.219
Phenyl-alanine	0.04 [0.04–0.05]	0.04 [0.03–0.04]	-0.009	[-0.017, -0.001]	**0.037**	0.226
Valine	0.24 [0.21–0.27]	0.23 [0.21–0.27]	-0.031	[-0.061, -0.001]	**0.040**	0.236
Tyrosine	0.05 [0.04–0.06]	0.05 [0.04–0.05]	-0.014	[-0.028, -0.000]	**0.048**	0.265

Values are medians with ranges. Effect(log2) is the difference on the log2 scale (lipedema - controls). Raw *p*-values were corrected using the Benjamini-Hochberg method for multiple testing to obtain FDR q-values. Bold values indicate statistically significant results.

The analyses revealed significantly lower fasting insulin (age-adjusted *p* = 0.032) and HOMA-IR values (age-adjusted *p* = 0.031) in the lipedema group compared to controls, indicating higher insulin sensitivity in this group. To investigate the relationship between insulin and body composition, we performed a correlation analysis. In the lipedema group, fasting insulin correlated strongly with waist circumference (Spearman’s *ρ* = 0.649, *p* = 0.012) and waist-to-height ratio (*ρ* = 0.636, *p* = 0.015), confirming its role as an indicator of central fat mass (**Supplementary Figures 1A, B**). There were no significant differences between the groups in HbA1c, triglycerides, or HDL cholesterol (**Table 2**). Sensitivity analyses excluding participants with type 2 diabetes confirmed that the observed metabolic differences remained consistent (**Supplementary Tables 7 and S8**).

In addition, the inflammatory markers CRP, IL-6, and TNF did not differ significantly between groups (**Table 2**). These findings highlight the importance of multivariate analytical approaches, as individual markers alone may not capture the subtle but clinically relevant metabolic alterations associated with lipedema.

### 3.2 NMR metabolomics reveals individual metabolic traits in patients with lipedema

**Figure 2:**
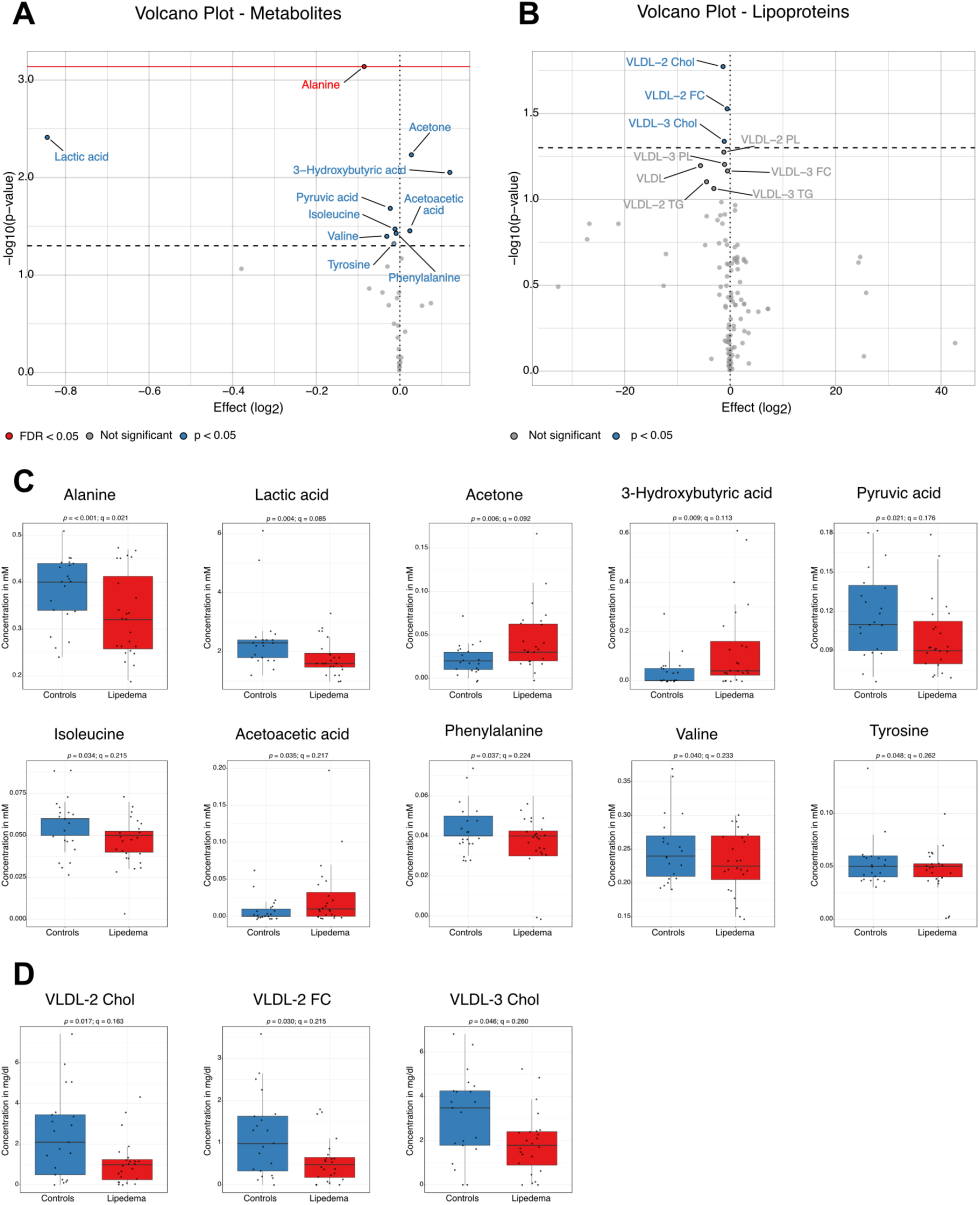
Single-metabolite and -lipoprotein NMR differences between lipedema and controls. **(A)** Volcano plot for all quantified metabolites. The grey dashed line indicates the nominal threshold (*p* = 0.05); the red horizontal line marks *p* = 0.001. Labeled points highlight metabolites with the strongest signals: Alanine remains significant after FDR correction (q<0.05; lower in lipedema), whereas blue labels denote nominal signals that did not pass FDR. **(B)** Volcano plot for all quantified lipoprotein measures. The red line marks the nominal threshold (*p* = 0.05). **(C)** Boxplots with individual data for representative metabolites (alanine, lactate, acetone, 3-hydroxybutyric acid, pyruvic acid, isoleucine, acetoacetic acid, phenylalanine, valine, tyrosine). **(D)** Boxplots with individual data for VLDL-2 cholesterol, VLDL-2 free cholesterol and VLDL-3 cholesterol. Boxes show median and IQR; points are individuals; n=21 controls (blue), n=24 lipedema (red). VLDL, very-low-density lipoprotein; FC, free cholesterol; Chol, total cholesterol in the indicated subfraction.

We next compared individual NMR metabolites between groups (**Table 3**; **Figures 2A, C**; full list in **Supplementary Table 1**). Alanine showed the most significant group difference, with lower levels observed in lipedema (median 0.32 *vs*. 0.40; *p* < 0.001; *q* = 0.021). Two central metabolites in glycolysis were also reduced in lipedema, specifically lactate (*p* = 0.004, *q* = 0.086) and pyruvate (*p* = 0.021, *q* = 0.178). Ketone bodies tended to be higher in lipedema, including acetone: (*p* = 0.006, *q* = 0.093), 3-hydroxybutyrate: *p* = 0.009, *q* = 0.114), and acetoacetate: (*p* = 0.035, *q* = 0.219).

To test whether these metabolic shifts were driven by anthropometric differences, we examined corresponding correlations with NMR metabolites. In the lipedema group, neither glycolysis markers nor ketone bodies showed a significant correlation with waist circumference, WHpR, or WHtR (all *p* > 0.05) (**Supplementary Figure 1C**). This suggests that the observed metabolic signature is not closely associated with these common anthropometric parameters.

Among the amino acids, slightly decreased levels were noted for isoleucine, phenylalanine, valine, and tyrosine (all *p* < 0.05). However, none of these changes remained significant after FDR correction (all *q*>0.20). Overall, single-metabolite testing identified alanine as the only metabolite that remained significant after FDR correction, whereas lactate, pyruvate, ketone bodies, and selected amino acids showed nominal group differences that did not consistently survive correction for multiple testing and should therefore be interpreted as exploratory trends. To address potential confounding by metformin-treated type 2 diabetes in the control group, we repeated the age-adjusted ANCOVA on individual metabolites after excluding these four participants (n=17 controls *vs*. n=24 lipedema) (**Supplementary Table 8**). All nominal group differences remained at *p* < 0.05 with essentially unchanged effect estimates and directions. As expected from the reduced sample size, no metabolite retained significance after FDR correction (alanine: *q* = 0.070).

Furthermore, blood lipoprotein levels and lipoprotein composition did not differ between the groups (**Supplementary Table 2**). At the subfraction level, however, a consistent pattern of lower VLDL components was observed in women with lipedema (**Figures 2B, D**). VLDL total particle count tended to be lower (*p* = 0.138; *q* = 0.45), with specifically lower cholesterol and free cholesterol levels in medium VLDL subfractions (VLDL-2 cholesterol: *p* = 0.017, *q* = 0.165; VLDL-3 cholesterol: *p* = 0.046, *q* = 0.260 and VLDL-2 free cholesterol: *p* = 0.030, *q* = 0.217). While these effects did not reach formal statistical significance after FDR correction, results indicate a consistent trend toward lower VLDL lipid load in lipedema. Overall, LDL and HDL subfractions were comparable; minor shifts (e.g. slightly higher LDL-2/-3/-4 cholesterol and free cholesterol levels in lipedema) also remained below the FDR threshold. In conclusion, the data argue against a classic atherogenic lipid profile in lipedema, suggesting instead subtle shifts in the cholesterol content of VLDL subfractions.

### 3.3 DXA characterizes the lipedema phenotype by assessing regional fat distribution

**Figure 3:**
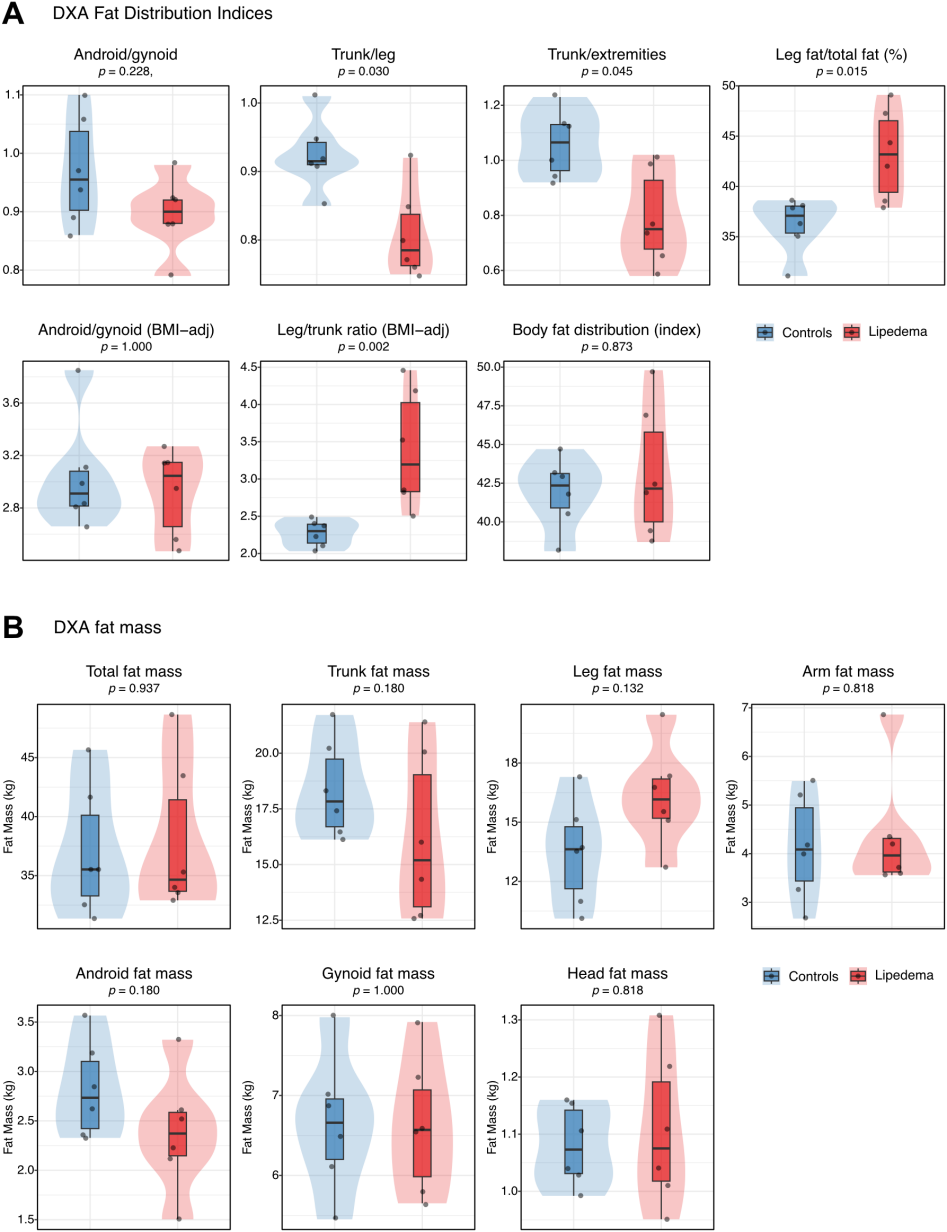
DXA fat mass and fat distribution indices in a matched subset of controls and lipedema patients. **(A)** DXA fat mass measurements for a matched subset of controls (blue, n=6) and lipedema patients (red, n=6). **(B)** DXA fat distribution indices for the same matched subset.

In the explorative dual-energy x-ray absorptiometry (DXA) sub-study (n = 12), a 1:1 matched set of 6 controls and 6 participants with lipedema was analyzed to minimize confounding by overall adiposity. Given the very small sample size, this analysis is presented descriptively, without correction for multiple testing and serves to illustrate the regional fat distribution pattern that motivates the composite Fat Distribution Index. In this tightly matched set, regional fat distribution varied clearly (**Figure 3A**). Specifically, DXA-derived distribution indices revealed a higher BMI-adjusted leg-to-trunk ratio in the lipedema group (*p* = 0.002). Congruently, leg fat mass as a proportion of total body fat was also greater in participants with lipedema (*p* = 0.015). Furthermore, both the trunk-to-leg fat ratio (*p* = 0.030) and the trunk-to-extremities ratio (*p* = 0.045) were lower in the lipedema group (**Figure 3B**). In contrast, other variables, including the android-to-gynoid ratio, trunk-to-extremities ratio, and overall regional fat masses, did not show significant group differences. Overall, these DXA findings corroborate a peripheral/gynoid predominance of adipose tissue in lipedema that persists independent of total fat mass.

### 3.4 Composite indices uncover the metabolic distinction of lipedema

**Figure 4:**
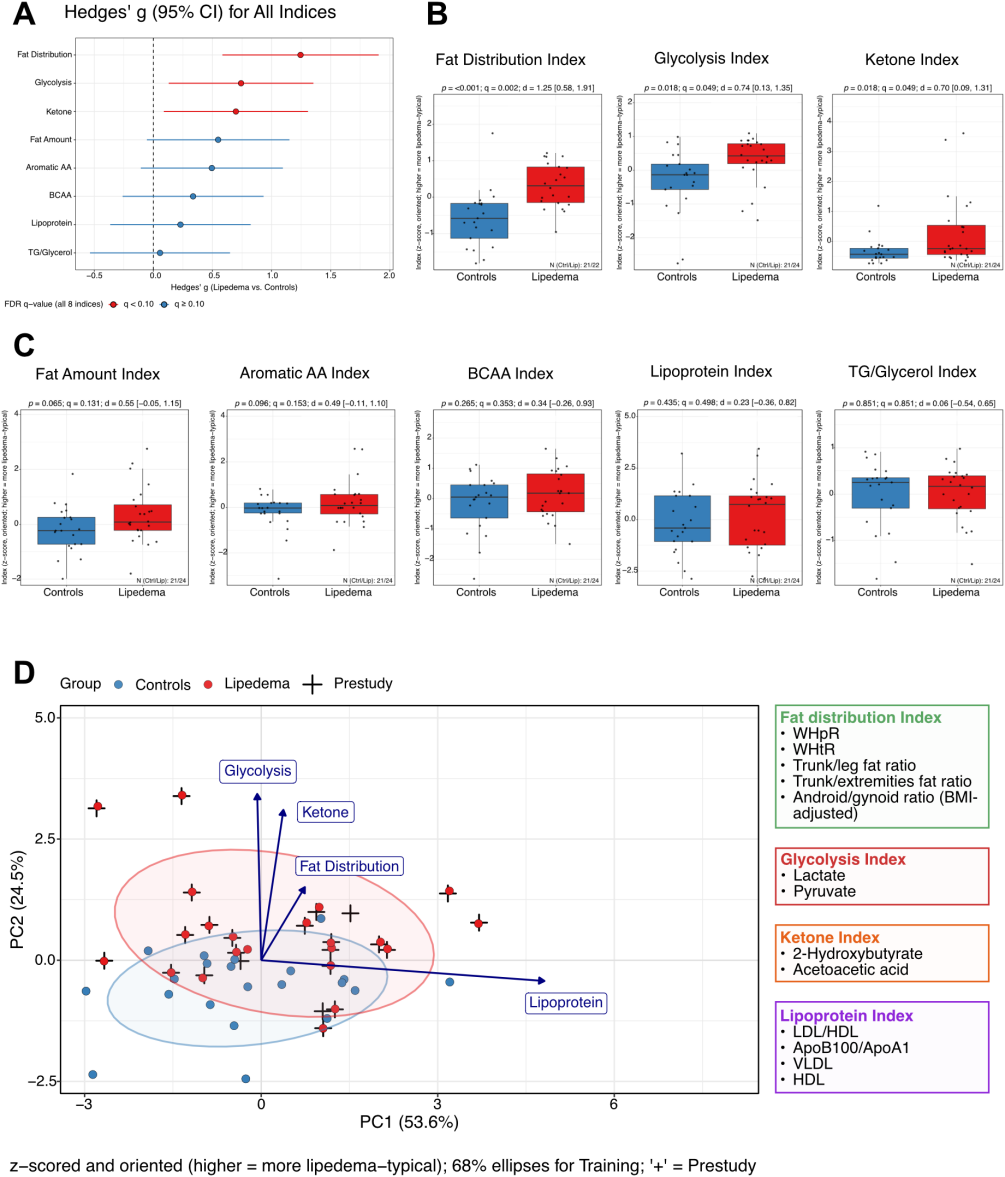
Group differences across composite indices. **(A)** Standardized mean differences (Hedges’ g; Lipedema *vs*. Controls) for all eight indices. Dots represent point estimates; horizontal lines show 95% confidence intervals. Red indicates FDR q-values < 0.05; blue denotes non-significant results (q ≥ 0.10). **(B, C)** Group comparisons of the indices (z-standardized; higher values = more lipedema-typical). Boxplots display individual values, Welch/Mann-Whitney *p*-values, FDR-adjusted q-values (corrected across four tests), and Hedges’ *g* with 95% confidence intervals. **(D)** PCA biplot based on the four oriented indices. Axis labels give explained variance. Filled circles represent training samples (red = lipedema, blue = controls). 68% ellipses visualize within-group dispersion. Crosses (+) denote the projected pre-study samples. Arrows show index loadings.

To characterize group differences beyond single analytes, we computed eight z-standardized metabolic and body composition indices. These indices capture broader physiological domains relevant to lipedema, such as fat distribution, energy metabolism, and amino acid profiles. A comprehensive overview of group differences across all indices is provided in **Figure 4A**. The largest and most statistically robust differences were observed for the Fat Distribution, Glycolysis, and Ketone indices, all of which showed strong effects and FDR-corrected *q*-values below 0.05. Additionally, moderate group differences were observed for the Branched-Chain Amino Acid (BCAA) and Aromatic Amino Acid indices, though these did not pass multiple testing correction.

For detailed group-level comparisons, we selected four indices based on statistical power and pathophysiological relevance to lipedema. The Fat Distribution Index was markedly elevated in the lipedema group compared to controls (*p* < 0.001, *q* < 0.001, Hedges’ *g* = 1.26 [0.58, 1.91]), reflecting characteristic adipose tissue distribution patterns. The Glycolysis Index was also significantly elevated in lipedema (*p* = 0.018, *q* = 0.025, Hedges’ *g* = 0.74 [0.13, 1.35]), suggesting altered glucose metabolism. The Ketone Index was similarly higher in the lipedema group (*p* = 0.018, *q* = 0.025, Hedges’ *g* = 0.70 [0.09, 1.31]), pointing to differences in lipid oxidation or fasting response (**Figure 4B**). In contrast, no significant group difference was found for the overall Lipoprotein Index (*p* = 0.541, *q* = 0.541, Hedges’ *g* = 0.18 [–0.41, 0.77]) (**Figure 4C**).

Principal component analysis (PCA) on the four oriented indices showed that the first two principal components (PC1 and PC2) explain a total of 78.1% of the total variance in the data (PC1: 53.6%, PC2: 24.5%) (**Figure 4D**, **Supplementary Figure 2**). The 68% ellipses in the PCA biplot show that although lipedema and control samples have an overlapping distribution, their group centers are separate, especially along the glycolysis/ketone axis (PC2). The first principal component (PC1), on the other hand, is mainly influenced by the lipoprotein index. The projection of the data from the non-fasting pre-study lipedema cohort into the same PCA space shows that these samples fall within the distribution of the lipedema cohort.

A multivariate comparison by PERMANOVA indicated an overall group difference (**Supplementary Table 3**; *F* = 4.04, *R²* = 0.09, *p* = 0.010). Leave-one-out (**Supplementary Table 4**) and bootstrap analyzes (**Supplementary Figure 3**) demonstrated stable effect sizes (*Δg* ≤ 0.05; bootstrap 95% CIs overlapped the original estimates). 82% of the non-fasted pre-study sera (18/22) clustered closer to the lipedema centroid than to controls (permutation *p* = 0.023) (**Supplementary Table 5**). These sensitivity analyzes further demonstrated that these group differences persist robustly. Age-adjusted ANCOVA suggested that the significant effects for the Glycolysis and Fat distribution indices were not confounded by age (**Supplementary Table 6**).

To further ensure that the observed group separation in the PCA was not driven by the higher fasting insulin or the presence of type 2 diabetes in the control group, sensitivity analyzes were performed. The univariate and multivariate group signals remained consistent after excluding participants with type 2 diabetes (**Supplementary Table 7, S8**).

## 4 Discussion

In this BMI-matched, fasting cohort of premenopausal women, we identified a multivariate signature that differed between women with lipedema and BMI-matched controls. Among the four preselected indices used for PCA, the Fat Distribution Index (Hedges *g* = 1.26), Glycolysis Index (*g* = 0.74), and Ketone Index (*g* = 0.70) were significantly higher in lipedema, whereas the Lipoprotein Index showed no group difference. The first two principal components explained 78% of the overall variance, and permutation MANOVA indicated a significant overall group effect. Notably, 82% of serum NMR profiles obtained from non-fasted lipedema patients in a previous pilot study (13) still clustered with the lipedema centroid rather than with the control centroid. Fasting insulin correlated with waist circumference and waist-to-height ratio in the group with lipedema, confirming its role as a marker of central adiposity, and showed an inverse association with ketone bodies. In contrast, anthropometric parameters themselves did not correlate with glycolysis or ketogenesis, suggesting that the metabolic signature of lipedema is not merely a consequence of fat distribution. At the single-metabolite level, only alanine remained significant after FDR correction. However, nominal group differences in lactate and pyruvate (lower in lipedema) together with nominally higher ketone bodies (3-hydroxybutyrate, acetoacetate, acetone) are also compatible with a shift from glycolysis towards fatty acid oxidation. The overall pattern persisted after adjustment for age and after exclusion of metformin-treated controls with type 2 diabetes, supporting the concept that lipedema represents a metabolically distinguishable phenotype (5, 16). However, this interpretation should be regarded as exploratory pending confirmation in larger and longitudinal cohorts.

Several of the affected metabolites converge on hepatic substrate handling. Alanine is a key substrate for hepatic gluconeogenesis via the glucose-alanine cycle (17, 18) and the lower systemic alanine in lipedema is consistent with altered flux through this pathway (19). The accompanying nominal reductions in medium-sized VLDL subfractions and nominal increases in ketone bodies are compatible with a coordinated hepatic re-routing of substrates between gluconeogenesis, lipoprotein assembly, and ketogenesis. This creates an apparent paradox: why do patients accumulate fat despite a systemic preference for fatty acid oxidation? Part of the answer could lie in the local pathophysiology of the adipose tissue itself. Histological studies show that lipedema fat is uniquely resistant to breakdown due to hypertrophic adipocytes, chronic inflammation, and fibrosis (20). This local tissue resistance could act as a physical barrier to fat mobilization, which may explain why patients have a lean upper body while the affected lipedema fat remains difficult to reduce. At the systemic level, however, the metabolic pattern itself could have two distinct origins: a maladaptive disturbance of glycolytic or gluconeogenic function, or a constitutive shift in substrate preference toward fatty acid oxidation, representing a functional metabolic adaptation rather than a dysfunction. The present cross-sectional design cannot distinguish these possibilities, and mechanistic confirmation of hepatic involvement would require dedicated liver-specific assessment.

These mechanistic questions also intersect with current clinical practice. Carbohydrate-restricted dietary protocols (especially ketogenic and low-carbohydrate, high-fat (LCHF) diets) have been proposed as therapeutic approaches for lipedema, in part because they directly shift systemic substrate use toward fatty acid oxidation, the very pattern we observe here in the fasting state. A recent systematic review (21) and various controlled trials suggest that these interventions can reduce body weight and fat mass and may also improve patient-reported outcomes, such as pain and quality of life, in some patients (22–25). In our cohort, no participant reported following such a regimen at the time of recruitment. Rather, the convergence between the fasting metabolic profile we describe and the substrate shift induced by carbohydrate-restricted diets raises the hypothesis that the clinical efficacy of these interventions in lipedema may build upon and reinforce a pre-existing systemic substrate preference toward fatty acid oxidation, rather than reverse a metabolic dysfunction. Whether this represents a true mechanistic alignment or coincidental overlap would need to be tested in prospective interventional studies that combine dietary monitoring with serial metabolomic profiling.

This study has several limitations that must be considered when interpreting the results. First, the inclusion of participants with metformin-treated type 2 diabetes in the control group is a potential confounder. As detailed in the Results section, the metabolic signature remained after excluding metformin-treated participants. Also, it is a cross-sectional study, which limits the derivation of causal relationships. Longitudinal studies would be necessary to track the development of metabolic and body composition-related changes over time and to establish causal relationships. It should be also noted that while mass spectrometry-based methods generally offer superior sensitivity for low-abundance metabolites, nuclear magnetic resonance (NMR-) spectroscopy was employed in this study as a robust platform for its high reproducibility and ability to provide an integrated quantification of both small-molecule metabolites and detailed lipoprotein subfractions without extensive sample preparation. While this approach entails a trade-off in absolute sensitivity, the high-throughput nature and cost-efficiency of NMR provide a promising framework for future clinical screening and large-scale diagnostic applications, where scalability is essential. Diet was captured by a single qualitative item only, and the exact fasting duration was not recorded. However, no participant reported a ketogenic or carbohydrate-restricted regimen, and the metabolic signature persisted in the non-fasted pilot cohort, arguing against a primarily diet-driven effect. Another important limitation is the non-fasting status of the previously published pilot cohort and the inter-individual variability in the metabolic response to fasting duration (13). Metabolic markers such as glucose, triglycerides, and certain amino acids are strongly influenced by food intake (26, 27). Therefore, direct comparisons of this group with the fasting cohorts are exploratory in nature and must be interpreted with caution. In addition, the integration of additional data sources (such as genetics, hormonal influences and imaging techniques, providing more detailed information about tissue properties, and a comprehensive correlation of clinical symptoms) is crucial and offers potential for further research.

## 5 Conclusion

In summary, our findings provide first evidence that lipedema is linked to a unique combination of gynoid fat distribution and a distinct metabolic trait that significantly differs from BMI-matched female controls without lipedema. The observed changes in glycolysis and ketone metabolism may indicate shifts in energy metabolism, with potential implications for targeted dietary strategies. These results highlight the idea of lipedema as a unique clinical condition and support the development of specific diagnostic tools based on multivariate metabolic signatures. Future longitudinal studies should determine whether these metabolic patterns remain consistent over time, respond to treatment, or predict clinical outcomes.

## Data Availability

The datasets presented in this article are not readily available because Data may be made available from the corresponding author upon reasonable request. Requests to access the datasets should be directed to SK, sally.kempa@ukr.de.
