# Supplementary material for: Distinct systemic metabolic signatures in premenopausal women with lipedema revealed by composite indices
Source: Front Endocrinol (Lausanne). 2026 Jun 5;17:1857893. doi: 10.3389/fendo.2026.1857893 (PMC13278978; doi:10.3389/fendo.2026.1857893)

Supplementary Material

**Supplementary Methods**

The statistical analyses were performed using R (version 4.5.1; R Core Team, 2025) within the RStudio (version 2025.05.1; Posit Software, PBC, Boston, MA, USA) platform. Continuous variables were assessed for normality with the Shapiro–Wilk test. Normally distributed data are reported as mean ± SD and compared with an independent samples t-test (with Welch's correction applied if variances were unequal, as assessed by Levene's test). In contrast, non-Gaussian data are summarized as median [IQR] and analyzed with the Mann–Whitney *U*-test. For a few variables, namely fasting insulin, HOMA-IR, IL-6, TNF, and neck circumference, there were >10% missing values. These were addressed by excluding the subjects from the analysis of these specific variables, as indicated in **Table 2** (main manuscript). To account for the significant age difference between groups, all primary comparisons were repeated in an Analysis of Covariance (ANCOVA) framework, adjusting for age (**Supplementary** **Tables S1, S2, S6, S7 and S8**). For variables with substantial skewness or non-normality of residuals in the initial ANCOVA model, data were transformed prior to ANCOVA. A natural logarithm transformation was applied to strictly positive variables, while otherwise a rank-normal transformation (inverse normal transformation of ranks) was used. The Benjamini-Hochberg procedure was used to control for false-discovery rate (FDR). An FDR-adjusted q-value of *q* < 0.05 was considered significant. Nominal p-values (*p* < 0.05) that did not survive FDR correction are reported and interpreted as exploratory only, pending confirmation in larger studies. Effect sizes are given as Hedges’ g with 95 % confidence intervals (CI). Correlations between metabolic and anthropometric variables within the lipedema group were evaluated using Spearman's rank correlation coefficient (*ρ*) (**Supplementary Figure S1**).

The eight composite indices were z-standardized and used for pairwise group comparisons and as input for principal-component analysis (PCA). The latter was performed on four indices (Fat Distribution, Glycolysis, Ketone, Lipoprotein), after centering but without additional scaling, since the variables were already expressed as *z*-scores. Scores from a previously published, non-fasted lipedema cohort measured by the same method (13) were projected into the same PC space to assess external consistency. Overall group separation was tested with a distance-based permutation MANOVA (**Supplementary Table S3**).

Robustness of the findings was examined in five complementary ways. First, each index was recalculated while sequentially omitting one of its constituent variables (leave-one-component-out), and the resulting changes in Hedges’ g were documented (**Supplementary Table S4**). Second, bias-corrected and accelerated bootstrap confidence intervals were generated from 2000 resamples for every index-wise group effect (**Supplementary Figure S3**). Third, projection strength of the pre-study samples was quantified by the Mahalanobis distance to the lipedema and control centroids in PC1–PC2 space (**Supplementary Table S5**). Fourth, to ensure that the observed metabolic differences were not driven by the four control participants with metformin-treated type 2 diabetes, a sensitivity analysis was conducted in which all composite indices and individual NMR metabolites were re-evaluated after excluding these individuals (**Supplementary Tables S7 and S8**). Fifth, to distinguish between the intrinsic effects of lipedema and the influence of systemic hyperinsulinemia, ANCOVA models were additionally adjusted for fasting insulin (**Supplementary Table S7**).

**Supplementary Table S1.** Age-adjusted between-group comparisons of NMR-derived metabolites (ANCOVA, adjusted for age)

| **Variable** | **Controls** (Mean±SD or Median(Range)) | **Lipedema** (Mean±SD or Median(Range)) | **Effect** (log2, age-adj.) | **95% CI** (age-adj.) | ***p*-value** (age-adj.) | **FDR q-value** (age-adj.) | |
| --- | --- | --- | --- | --- | --- | --- | --- |
| ethanol | 0.00 [0.00–0.00] | 0.00 [0.00–0.00] | -0.003 | [-0.012, 0.007] | 0.573 | 0.777 | |
| trimethylamine-N-oxide | 0.00 [0.00–0.00] | 0.00 [0.00–0.00] | 0.000 | [0.000, 0.000] | | |  |
| 2-aminobutyric acid | 0.00 [0.00–0.00] | 0.00 [0.00–0.01] | 0.003 | [-0.014, 0.020] | 0.701 | 0.859 | |
| **alanine** | **0.40 [0.34–0.44]** | **0.32 [0.26–0.41]** | **-0.085** | **[-0.133, -0.038]** | **<0.001** | **0.021** | |
| asparagine | 0.00 [0.00–0.00] | 0.00 [0.00–0.00] | 0.000 | [0.000, 0.000] | | |  |
| creatine | 0.02 [0.02–0.04] | 0.02 [0.01–0.03] | -0.005 | [-0.017, 0.007] | 0.437 | 0.643 | |
| creatinine | 0.08 ± 0.02 | 0.08 ± 0.01 | -0.007 | [-0.017, 0.003] | 0.172 | 0.469 | |
| glutamic acid | 0.00 [0.00–0.06] | 0.00 [0.00–0.06] | -0.014 | [-0.042, 0.014] | 0.315 | 0.578 | |
| glutamine | 0.62 ± 0.09 | 0.62 ± 0.12 | -0.042 | [-0.099, 0.016] | 0.151 | 0.469 | |
| glycine | 0.23 [0.19–0.27] | 0.23 [0.20–0.25] | -0.027 | [-0.068, 0.015] | 0.204 | 0.469 | |
| histidine | 0.07 [0.07–0.08] | 0.08 [0.07–0.08] | -0.001 | [-0.013, 0.011] | 0.866 | 0.948 | |
| **isoleucine** | **0.06 [0.05–0.06]** | **0.05 [0.04–0.05]** | **-0.012** | **[-0.022, -0.001]** | **0.034** | **0.217** | |
| leucine | 0.09 [0.08–0.11] | 0.10 [0.08–0.11] | -0.002 | [-0.018, 0.014] | 0.799 | 0.914 | |
| lysine | 0.15 [0.14–0.19] | 0.15 [0.13–0.19] | -0.030 | [-0.063, 0.004] | 0.082 | 0.358 | |
| methionine | 0.00 [0.00–0.05] | 0.00 [0.00–0.06] | -0.004 | [-0.023, 0.016] | 0.691 | 0.852 | |
| N,N-dimethylglycine | 0.00 [0.00–0.00] | 0.00 [0.00–0.00] | 0.000 | [0.000, 0.000] | | |  |
| ornithine | 0.00 [0.00–0.02] | 0.00 [0.00–0.03] | -0.001 | [-0.012, 0.010] | 0.874 | 0.948 | |
| phenylalanine | 0.04 [0.04–0.05] | 0.04 [0.03–0.04] | -0.009 | [-0.017, -0.001] | **0.037** | 0.226 | |
| proline | 0.00 [0.00–0.24] | 0.00 [0.00–0.00] | -0.073 | [-0.171, 0.024] | 0.137 | 0.446 | |
| sarcosine | 0.00 [0.00–0.00] | 0.00 [0.00–0.00] | 0.000 | [0.000, 0.000] | | |  |
| threonine | 0.09 [0.00–0.11] | 0.08 [0.05–0.12] | -0.002 | [-0.044, 0.041] | 0.941 | 0.976 | |
| tyrosine | 0.05 [0.04–0.06] | 0.05 [0.04–0.05] | -0.014 | [-0.028, -0.000] | **0.048** | 0.265 | |
| valine | 0.24 [0.21–0.27] | 0.23 [0.21–0.27] | -0.031 | [-0.061, -0.001] | **0.040** | 0.236 | |
| 2-hydroxybutyric acid | 0.00 [0.00–0.00] | 0.00 [0.00–0.00] | 0.000 | [0.000, 0.000] | | |  |
| acetic acid | 0.03 [0.03–0.04] | 0.03 [0.03–0.04] | 0.001 | [-0.008, 0.010] | 0.781 | 0.909 | |
| citric acid | 0.19 [0.17–0.20] | 0.18 [0.16–0.21] | 0.013 | [-0.016, 0.041] | 0.381 | 0.618 | |
| formic acid | 0.03 [0.02–0.03] | 0.03 [0.02–0.03] | -0.004 | [-0.011, 0.004] | 0.329 | 0.585 | |
| **lactic acid** | **2.30 [1.80–2.40]** | **1.60 [1.48–1.95]** | **-0.844** | **[-1.400, -0.287]** | **0.004** | **0.086** | |
| succinic acid | 0.00 [0.00–0.00] | 0.00 [0.00–0.00] | 0.004 | [-0.000, 0.008] | 0.068 | 0.312 | |
| choline | 0.00 [0.00–0.00] | 0.00 [0.00–0.00] | 0.000 | [0.000, 0.000] | | |  |
| 2-oxoglutaric acid | 0.00 [0.00–0.00] | 0.00 [0.00–0.00] | -0.002 | [-0.005, 0.001] | 0.151 | 0.469 | |
| **3-hydroxybutyric acid** | **0.00 [0.00–0.05]** | **0.04 [0.02–0.16]** | **0.120** | **[0.032, 0.207]** | **0.009** | **0.114** | |
| **acetoacetic acid** | **0.00 [0.00–0.01]** | **0.01 [0.00–0.03]** | **0.024** | **[0.002, 0.046]** | **0.035** | **0.219** | |
| **acetone** | **0.02 [0.01–0.03]** | **0.03 [0.02–0.06]** | **0.028** | **[0.008, 0.047]** | **0.006** | **0.093** | |
| **pyruvic acid** | **0.11 [0.09–0.14]** | **0.09 [0.08–0.11]** | **-0.023** | **[-0.042, -0.004]** | **0.021** | **0.178** | |
| D-galactose | 0.00 [0.00–0.00] | 0.00 [0.00–0.00] | 0.000 | [0.000, 0.000] | | |  |
| glucose | 4.86 ± 0.83 | 4.62 ± 0.53 | -0.379 | [-0.814, 0.056] | 0.086 | 0.362 | |
| glycerol | 0.00 [0.00–0.14] | 0.11 [0.00–0.22] | 0.053 | [-0.030, 0.136] | 0.206 | 0.469 | |
| dimethylsulfone | 0.00 [0.00–0.00] | 0.00 [0.00–0.00] | 0.074 | [-0.039, 0.188] | 0.194 | 0.469 | |

**Supplementary Table S2.** Age-adjusted between-group comparisons of NMR-derived lipoprotein subfractions (ANCOVA, adjusted for age)

| **Variable** | **Controls** (Mean±SD or Median(Range)) | **Lipedema** (Mean±SD or Median(Range)) | **Effect** (log2, age-adj.) | **95% CI** (age-adj.) | ***p*-value** (age-adj.) | **FDR q-value** (age-adj.) |
| --- | --- | --- | --- | --- | --- | --- |
| TG | 116.08 [62.45–134.05] | 89.64 [70.56–113.66] | -27.126 | [-66.407, 12.155] | 0.171 | 0.469 |
| Chol | 185.12 ± 29.10 | 184.05 ± 31.38 | 0.388 | [-19.486, 20.261] | 0.969 | 0.982 |
| LDL | 102.15 ± 28.30 | 108.80 ± 27.58 | 7.167 | [-11.145, 25.479] | 0.434 | 0.643 |
| HDL | 51.89 ± 9.15 | 54.14 ± 10.23 | 2.652 | [-3.731, 9.035] | 0.406 | 0.634 |
| ApoA1 | 143.52 ± 17.84 | 144.26 ± 22.74 | 3.489 | [-9.833, 16.810] | 0.600 | 0.802 |
| ApoA2 | 30.40 ± 3.41 | 29.98 ± 4.19 | -0.334 | [-2.859, 2.190] | 0.790 | 0.910 |
| ApoB100 | 78.64 ± 17.62 | 78.42 ± 20.00 | 1.396 | [-10.948, 13.741] | 0.821 | 0.924 |
| LDL/HDL | 2.03 ± 0.65 | 2.09 ± 0.67 | 0.066 | [-0.369, 0.502] | 0.760 | 0.905 |
| ApoB100/ApoA1 | 0.56 ± 0.14 | 0.55 ± 0.16 | 0.002 | [-0.097, 0.101] | 0.973 | 0.982 |
| ApoB Particles | 1429.93 ± 320.29 | 1425.80 ± 363.62 | 25.379 | [-199.069, 249.827] | 0.821 | 0.924 |
| VLDL Particles | 142.23 ± 63.65 | 113.80 ± 44.90 | -26.705 | [-62.393, 8.982] | 0.138 | 0.446 |
| IDL Particles | 87.66 ± 40.88 | 71.97 ± 36.65 | -12.623 | [-37.869, 12.623] | 0.319 | 0.578 |
| LDL Particles | 1186.78 ± 323.96 | 1213.70 ± 323.78 | 42.684 | [-169.421, 254.788] | 0.687 | 0.852 |
| LDL-1 Particles | 219.92 ± 57.83 | 214.25 ± 56.26 | -3.550 | [-40.908, 33.808] | 0.849 | 0.940 |
| LDL-2 Particles | 168.50 ± 64.73 | 188.85 ± 57.00 | 24.673 | [-15.003, 64.349] | 0.216 | 0.469 |
| LDL-3 Particles | 191.92 ± 60.91 | 215.00 ± 62.82 | 24.352 | [-16.280, 64.984] | 0.233 | 0.469 |
| LDL-4 Particles | 180.33 ± 74.63 | 210.40 ± 91.62 | 25.801 | [-29.296, 80.899] | 0.350 | 0.596 |
| LDL-5 Particles | 170.31 ± 87.41 | 175.31 ± 90.17 | 3.547 | [-54.776, 61.870] | 0.903 | 0.959 |
| LDL-6 Particles | 219.50 [205.05–277.72] | 208.09 [151.73–241.87] | -32.630 | [-98.380, 33.120] | 0.322 | 0.578 |
| VLDL TG | 66.24 [39.73–91.46] | 52.22 [39.99–75.97] | -21.198 | [-49.539, 7.142] | 0.139 | 0.446 |
| IDL TG | 9.45 [2.87–15.04] | 6.53 [3.58–10.88] | -4.732 | [-11.809, 2.345] | 0.184 | 0.469 |
| LDL TG | 16.28 [14.24–21.39] | 15.62 [12.56–20.47] | -0.989 | [-5.554, 3.575] | 0.664 | 0.852 |
| HDL TG | 11.42 [8.92–16.25] | 10.66 [9.29–12.21] | -0.700 | [-4.041, 2.641] | 0.675 | 0.852 |
| VLDL | 18.45 ± 11.21 | 12.64 ± 6.72 | -5.629 | [-11.590, 0.332] | 0.064 | 0.304 |
| IDL | 11.71 ± 5.78 | 9.34 ± 4.98 | -2.031 | [-5.538, 1.477] | 0.249 | 0.484 |
| LDL | 102.15 ± 28.30 | 108.80 ± 27.58 | 7.167 | [-11.145, 25.479] | 0.434 | 0.643 |
| HDL | 51.89 ± 9.15 | 54.14 ± 10.23 | 2.652 | [-3.731, 9.035] | 0.406 | 0.634 |
| VLDL FC | 8.50 ± 4.42 | 6.58 ± 2.76 | -1.852 | [-4.232, 0.528] | 0.124 | 0.446 |
| IDL FC | 3.31 ± 1.57 | 2.63 ± 1.43 | -0.582 | [-1.562, 0.397] | 0.237 | 0.469 |
| LDL FC | 29.39 ± 7.40 | 31.45 ± 7.25 | 2.093 | [-2.710, 6.897] | 0.384 | 0.618 |
| HDL FC | 11.19 ± 2.59 | 12.01 ± 3.26 | 1.167 | [-0.759, 3.093] | 0.228 | 0.469 |
| VLDL PL | 21.09 ± 10.64 | 16.60 ± 6.73 | -4.460 | [-10.213, 1.293] | 0.125 | 0.446 |
| IDL PL | 6.66 ± 3.68 | 4.88 ± 2.42 | -1.658 | [-3.668, 0.353] | 0.104 | 0.411 |
| LDL PL | 59.90 ± 13.68 | 62.89 ± 14.09 | 3.455 | [-5.659, 12.569] | 0.449 | 0.654 |
| HDL PL | 76.48 [67.96–79.75] | 74.30 [69.93–90.80] | 2.705 | [-7.220, 12.631] | 0.585 | 0.788 |
| HDL ApoA1 | 141.74 [130.81–146.10] | 137.96 [132.44–160.01] | 5.354 | [-8.849, 19.558] | 0.451 | 0.654 |
| HDL ApoA2 | 30.72 ± 3.31 | 30.26 ± 4.10 | -0.398 | [-2.859, 2.063] | 0.746 | 0.893 |
| VLDL ApoB | 7.82 ± 3.50 | 6.26 ± 2.47 | -1.469 | [-3.431, 0.493] | 0.138 | 0.446 |
| IDL ApoB | 4.82 ± 2.25 | 3.96 ± 2.02 | -0.694 | [-2.083, 0.694] | 0.318 | 0.578 |
| LDL ApoB | 65.27 ± 17.82 | 66.75 ± 17.81 | 2.348 | [-9.318, 14.013] | 0.687 | 0.852 |
| VLDL-1 TG | 29.69 [19.72–48.08] | 28.11 [18.97–38.62] | -12.181 | [-31.402, 7.040] | 0.208 | 0.469 |
| VLDL-2 TG | 11.33 [5.33–16.18] | 7.79 [4.72–10.29] | -4.468 | [-9.475, 0.539] | 0.079 | 0.353 |
| VLDL-3 TG | 10.83 [4.95–13.59] | 6.52 [3.67–8.17] | -3.088 | [-6.636, 0.461] | 0.086 | 0.362 |
| VLDL-4 TG | 8.37 ± 3.67 | 7.32 ± 2.75 | -0.837 | [-2.937, 1.264] | 0.426 | 0.643 |
| VLDL-5 TG | 2.42 [2.11–3.07] | 2.87 [2.38–3.10] | 0.225 | [-0.276, 0.726] | 0.370 | 0.612 |
| VLDL-1 Chol | 4.87 [2.08–6.97] | 4.11 [2.50–5.66] | -2.400 | [-5.552, 0.753] | 0.132 | 0.446 |
| **VLDL-2 Chol** | **2.10 [0.50–3.45]** | **0.99 [0.25–1.25]** | **-1.344** | **[-2.433, -0.255]** | **0.017** | **0.165** |
| **VLDL-3 Chol** | **3.12 ± 1.94** | **1.87 ± 1.41** | **-1.117** | **[-2.213, -0.021]** | **0.046** | **0.263** |
| VLDL-4 Chol | 4.10 ± 2.19 | 3.18 ± 1.81 | -0.899 | [-2.208, 0.410] | 0.173 | 0.469 |
| VLDL-5 Chol | 1.29 ± 0.57 | 1.51 ± 0.46 | 0.190 | [-0.145, 0.526] | 0.259 | 0.494 |
| VLDL-1 FC | 2.67 [0.84–3.55] | 1.78 [1.19–2.72] | -1.007 | [-2.295, 0.281] | 0.122 | 0.446 |
| **VLDL-2 FC** | **0.98 [0.33–1.63]** | **0.48 [0.18–0.65]** | **-0.565** | **[-1.071, -0.059]** | **0.030** | **0.217** |
| VLDL-3 FC | 1.40 ± 1.01 | 0.84 ± 0.63 | -0.505 | [-1.049, 0.040] | 0.068 | 0.312 |
| VLDL-4 FC | 1.65 ± 1.00 | 1.30 ± 0.83 | -0.355 | [-0.956, 0.245] | 0.239 | 0.469 |
| VLDL-5 FC | 0.49 ± 0.29 | 0.52 ± 0.26 | 0.044 | [-0.134, 0.223] | 0.619 | 0.822 |
| VLDL-1 PL | 5.57 [3.21–8.42] | 4.85 [3.45–6.88] | -2.110 | [-5.233, 1.014] | 0.180 | 0.469 |
| VLDL-2 PL | 3.43 [1.52–4.42] | 2.20 [1.49–2.62] | -1.192 | [-2.401, 0.016] | 0.053 | 0.280 |
| VLDL-3 PL | 3.77 ± 1.98 | 2.63 ± 1.41 | -1.056 | [-2.170, 0.058] | 0.063 | 0.304 |
| VLDL-4 PL | 4.27 ± 1.81 | 3.64 ± 1.46 | -0.581 | [-1.653, 0.492] | 0.281 | 0.530 |
| VLDL-5 PL | 1.68 ± 0.64 | 1.87 ± 0.54 | 0.180 | [-0.206, 0.567] | 0.351 | 0.596 |
| LDL-1 TG | 5.13 [4.05–6.51] | 4.63 [3.69–6.31] | -0.420 | [-2.147, 1.308] | 0.626 | 0.822 |
| LDL-2 TG | 2.45 [2.00–3.19] | 2.21 [1.97–2.86] | -0.080 | [-0.687, 0.526] | 0.791 | 0.910 |
| LDL-3 TG | 2.41 [1.94–2.91] | 2.42 [2.09–2.84] | 0.056 | [-0.481, 0.593] | 0.834 | 0.934 |
| LDL-4 TG | 2.28 ± 1.18 | 2.26 ± 1.26 | -0.007 | [-0.809, 0.796] | 0.986 | 0.986 |
| LDL-5 TG | 2.10 [1.25–2.78] | 1.50 [0.90–2.04] | -0.367 | [-1.185, 0.452] | 0.371 | 0.612 |
| LDL-6 TG | 3.29 [2.68–5.63] | 3.00 [2.25–3.15] | -0.589 | [-1.468, 0.290] | 0.184 | 0.469 |
| LDL-1 Chol | 22.38 ± 5.83 | 22.12 ± 5.67 | -0.264 | [-4.033, 3.505] | 0.888 | 0.956 |
| LDL-2 Chol | 16.36 ± 7.12 | 18.68 ± 6.23 | 2.649 | [-1.713, 7.011] | 0.227 | 0.469 |
| LDL-3 Chol | 17.66 ± 6.58 | 20.19 ± 6.34 | 2.599 | [-1.636, 6.834] | 0.222 | 0.469 |
| LDL-4 Chol | 15.63 ± 7.25 | 19.03 ± 7.92 | 2.999 | [-1.988, 7.986] | 0.232 | 0.469 |
| LDL-5 Chol | 13.13 ± 7.27 | 14.13 ± 7.15 | 0.825 | [-3.902, 5.552] | 0.726 | 0.882 |
| LDL-6 Chol | 15.84 [11.98–19.50] | 14.21 [10.19–16.84] | -2.038 | [-6.473, 2.397] | 0.359 | 0.601 |
| LDL-1 FC | 6.50 ± 1.80 | 6.43 ± 1.70 | -0.034 | [-1.179, 1.111] | 0.953 | 0.976 |
| LDL-2 FC | 4.60 ± 2.25 | 5.32 ± 1.80 | 0.873 | [-0.448, 2.193] | 0.190 | 0.469 |
| LDL-3 FC | 4.82 ± 1.90 | 5.68 ± 1.61 | 0.913 | [-0.235, 2.061] | 0.116 | 0.442 |
| LDL-4 FC | 4.40 ± 1.73 | 5.40 ± 1.83 | 0.953 | [-0.218, 2.123] | 0.108 | 0.420 |
| LDL-5 FC | 3.52 ± 1.67 | 4.02 ± 1.69 | 0.471 | [-0.631, 1.573] | 0.393 | 0.626 |
| LDL-6 FC | 4.17 [3.23–5.15] | 3.76 [3.19–4.60] | -0.215 | [-1.181, 0.750] | 0.655 | 0.852 |
| LDL-1 PL | 13.18 ± 3.02 | 12.96 ± 3.13 | -0.161 | [-2.181, 1.859] | 0.873 | 0.948 |
| LDL-2 PL | 9.39 ± 3.57 | 10.54 ± 3.06 | 1.328 | [-0.834, 3.490] | 0.222 | 0.469 |
| LDL-3 PL | 10.01 ± 3.19 | 11.32 ± 3.13 | 1.386 | [-0.684, 3.457] | 0.184 | 0.469 |
| LDL-4 PL | 9.06 ± 3.53 | 10.70 ± 4.10 | 1.440 | [-1.078, 3.959] | 0.255 | 0.491 |
| LDL-5 PL | 7.60 ± 3.62 | 8.07 ± 3.68 | 0.410 | [-1.989, 2.809] | 0.732 | 0.882 |
| LDL-6 PL | 9.33 [7.73–11.05] | 8.77 [6.80–10.20] | -0.886 | [-3.068, 1.297] | 0.417 | 0.637 |
| LDL-1 ApoB | 12.09 ± 3.18 | 11.78 ± 3.09 | -0.196 | [-2.250, 1.859] | 0.848 | 0.940 |
| LDL-2 ApoB | 9.27 ± 3.56 | 10.39 ± 3.13 | 1.357 | [-0.825, 3.539] | 0.217 | 0.469 |
| LDL-3 ApoB | 10.55 ± 3.35 | 11.82 ± 3.46 | 1.340 | [-0.895, 3.575] | 0.233 | 0.469 |
| LDL-4 ApoB | 9.92 ± 4.10 | 11.57 ± 5.04 | 1.418 | [-1.612, 4.448] | 0.350 | 0.596 |
| LDL-5 ApoB | 9.37 ± 4.81 | 9.64 ± 4.96 | 0.196 | [-3.012, 3.403] | 0.903 | 0.959 |
| LDL-6 ApoB | 12.07 [11.28–15.27] | 11.45 [8.34–13.30] | -1.793 | [-5.409, 1.823] | 0.323 | 0.578 |
| HDL-1 TG | 4.06 [2.27–5.01] | 3.35 [2.66–4.45] | -0.337 | [-1.946, 1.272] | 0.675 | 0.852 |
| HDL-2 TG | 1.83 [1.35–2.76] | 1.85 [1.58–2.15] | -0.017 | [-0.663, 0.629] | 0.958 | 0.977 |
| HDL-3 TG | 2.25 [1.84–3.11] | 2.04 [1.68–2.45] | -0.042 | [-0.659, 0.575] | 0.891 | 0.956 |
| HDL-4 TG | 3.86 ± 1.14 | 3.53 ± 1.06 | -0.220 | [-0.933, 0.493] | 0.536 | 0.747 |
| HDL-1 Chol | 17.11 [12.04–21.64] | 16.68 [12.39–20.86] | -0.162 | [-4.663, 4.340] | 0.943 | 0.976 |
| HDL-2 Chol | 6.94 [6.14–7.73] | 8.24 [6.93–9.77] | 0.860 | [-0.549, 2.268] | 0.225 | 0.469 |
| HDL-3 Chol | 9.34 ± 1.82 | 10.08 ± 2.25 | 0.833 | [-0.517, 2.184] | 0.220 | 0.469 |
| HDL-4 Chol | 17.54 ± 4.47 | 18.55 ± 2.05 | 0.669 | [-1.542, 2.880] | 0.545 | 0.753 |
| HDL-1 FC | 3.78 [2.87–4.87] | 3.74 [3.14–5.27] | 0.080 | [-0.935, 1.095] | 0.874 | 0.948 |
| HDL-2 FC | 1.61 [1.34–1.93] | 1.91 [1.35–2.15] | 0.121 | [-0.235, 0.477] | 0.497 | 0.697 |
| HDL-3 FC | 1.88 ± 0.43 | 2.01 ± 0.55 | 0.143 | [-0.184, 0.470] | 0.383 | 0.618 |
| HDL-4 FC | 3.11 ± 0.96 | 3.45 ± 0.65 | 0.317 | [-0.215, 0.849] | 0.236 | 0.469 |
| HDL-1 PL | 20.17 [15.38–25.35] | 19.73 [15.93–27.29] | -0.282 | [-6.377, 5.814] | 0.926 | 0.968 |
| HDL-2 PL | 11.85 [10.73–13.59] | 12.55 [11.19–16.42] | 1.002 | [-1.442, 3.447] | 0.413 | 0.634 |
| HDL-3 PL | 15.48 [14.65–17.06] | 16.38 [13.75–19.14] | 0.970 | [-1.388, 3.327] | 0.411 | 0.634 |
| HDL-4 PL | 26.18 ± 4.03 | 27.06 ± 3.54 | 0.702 | [-1.772, 3.176] | 0.570 | 0.777 |
| HDL-1 ApoA1 | 20.92 [15.90–29.00] | 22.88 [16.59–31.89] | -0.086 | [-9.074, 8.902] | 0.985 | 0.986 |
| HDL-2 ApoA1 | 18.35 [16.12–20.98] | 18.57 [16.05–22.53] | 0.395 | [-2.866, 3.656] | 0.808 | 0.920 |
| HDL-3 ApoA1 | 25.88 [23.37–28.45] | 26.25 [23.47–30.73] | 1.879 | [-1.757, 5.515] | 0.303 | 0.567 |
| HDL-4 ApoA1 | 69.86 ± 9.86 | 72.02 ± 8.21 | 2.011 | [-3.903, 7.925] | 0.496 | 0.697 |
| HDL-1 ApoA2 | 2.31 [1.89–3.45] | 2.46 [1.86–3.17] | -0.280 | [-1.245, 0.684] | 0.560 | 0.770 |
| HDL-2 ApoA2 | 3.45 [3.10–4.76] | 3.33 [2.90–4.27] | -0.133 | [-0.795, 0.529] | 0.688 | 0.852 |
| HDL-3 ApoA2 | 5.87 [5.60–6.70] | 5.88 [5.39–7.04] | -0.042 | [-0.933, 0.848] | 0.924 | 0.968 |
| HDL-4 ApoA2 | 17.48 ± 3.43 | 17.37 ± 2.52 | -0.412 | [-2.350, 1.526] | 0.670 | 0.852 |

**Supplementary Table S3**. PERMANOVA of group separation based on the four selected composite indices (Fat Distribution, Glycolysis, Ketone, Lipoprotein)

| **Test** | **R^2^** | **p-value** |
| --- | --- | --- |
| PERMANOVA | 0.090 | 0.0101 |

**Supplementary Table S4**. Leave-one-out analysis (LOOA) for composite indices

| **Index** | **Component removed** | **Hedges g** | **95% CI Lower** | **95% CI Upper** |
| --- | --- | --- | --- | --- |
| Fat Distribution Index | Waist-to-hip ratio (WHpR) | 0.63 | -0.19 | 1.45 |
|  | Trunk/leg fat ratio | 0.63 | -0.22 | 1.48 |
|  | Android/gynoid ratio | 1.11 | 0.23 | 1.99 |
|  | Android/gynoid ratio (BMI-adj.) | 0.47 | -0.37 | 1.32 |
| Glycolysis Index | Lactic acid | 0.63 | 0.02 | 1.23 |
|  | Pyruvic acid | 0.68 | 0.07 | 1.29 |
| Ketone Index | Acetoacetic acid | -0.69 | -1.29 | -0.08 |
|  | 3-Hydroxybutyric acid | -0.51 | -1.11 | 0.09 |
| Lipoprotein Index | ApoB100/ApoA1 ratio | 0.15 | -0.44 | 0.75 |
|  | LDL/HDL ratio | 0.21 | -0.38 | 0.81 |
|  | VLDL particle count | -0.18 | -0.77 | 0.41 |
|  | HDL | 0.18 | -0.41 | 0.77 |

**Supplementary Table S5**. Mahalanobis distance permutation test for clustering of previously published pilot cohort (13) samples.

| **Test** | **Prestudy samples closer to Lipedema** | **Total Prestudy samples** | **Permutation p-value** |
| --- | --- | --- | --- |
| Mahalanobis Permutation Test | 18 | 22 | 0.023 |

**Supplementary Table S6**. Age-adjusted ANCOVA results for the four composite indices used in the principal component analysis.

| **Index** | **Coefficient (beta)** | **t-value** | **p-value** |
| --- | --- | --- | --- |
| Fat Distribution Index | 0.97 | 3.56 | **<0.001** |
| Glycolysis Index | 0.91 | 2.63 | **0.012** |
| Ketone index | 0.76 | 1.92 | 0.062 |
| Lipoprotein Index | 0.26 | 0.49 | 0.628 |

**Supplementary Table S7.** Sensitivity analysis comparing women with lipedema (n=24) and BMI-matched controls without metformin-treated type 2 diabetes (n=17). Effect sizes are reported as Hedges’ g with 95% confidence intervals. P-values are provided for models adjusted for age and fasting insulin, respectively.

| **Index** | **Effect Size (Hedges' g)** | **95% confidence interval** | **p-value (Age-adjusted)** | **p-value (Insulin-adjusted)** |
| --- | --- | --- | --- | --- |
| **Fat distribution Index** | 1.14 | [0.45, 1.83] | 0.002 | **0.093** |
| **Ketone Index** | 0.70 | [0.05, 1.34] | 0.011 | **0.071** |
| **Glycolysis Index** | 0.64 | [0.01, 1.28] | 0.017 | **0.018** |
| **Aromatic amino acid Index** | 0.61 | [0.03, 1.26] | 0.009 | 0.204 |
| **Fat amount Index** | 0.50 | [-0.14, 1.13] | 0.095 | 0.403 |
| **Branched-chain amino acid Index** | 0.38 | [-0.25, 1.02] | 0.058 | 0.346 |
| **Lipoprotein Index** | 0.26 | [-0.37, 0.89] | 0.591 | 0.879 |
| **Triglycerides/Glycerol Index** | 0.04 | [-0.59, 0.67] | 0.877 | 0.876 |

**Supplementary Table S8.** Sensitivity analysis of individual NMR metabolites comparing women with lipedema (n=24) and BMI-matched controls without metformin-treated type 2 diabetes (n=17). Effect estimates and p-values are from age-adjusted ANCOVA models. P-values are sorted in ascending order.

| **Metabolite** | **Effect (Lipedema − Control)** | **Standard error** | **p-value (Age-adjusted)** | **FDR q-value** |
| --- | --- | --- | --- | --- |
| **Alanine** | −0.085 | 0.016 | **0.003** | 0.070 |
| **Acetone** | +0.029 | 0.007 | **0.009** | 0.164 |
| **3-Hydroxybutyric acid** | +0.127 | 0.030 | **0.013** | 0.164 |
| **Phenylalanine** | −0.011 | 0.003 | **0.014** | 0.164 |
| **Lactic acid** | −0.603 | 0.149 | **0.017** | 0.164 |
| **Tyrosine** | −0.019 | 0.005 | **0.017** | 0.164 |
| **Valine** | −0.038 | 0.010 | **0.022** | 0.195 |
| **Acetoacetic acid** | +0.027 | 0.007 | **0.032** | 0.239 |
| **Isoleucine** | −0.012 | 0.004 | **0.044** | 0.290 |
| **Pyruvic acid** | −0.022 | 0.007 | **0.046** | 0.297 |

**Supplementary Figure S1.** Correlation of fasting insulin with measures of central obesity in the lipedema group (n=14). Scatter plots illustrating the significant positive associations between fasting insulin and (A) waist circumference (Spearman's *ρ* = 0.649, *p* = 0.012) and (B) waist-to-height ratio (Spearman's *ρ* = 0.636, *p* = 0.015). (C) Spearman correlation matrix of metabolic and anthropometric variables. Color intensity indicates the strength of the correlation (orange for positive, blue for negative). Non-significant correlations *(p* > 0.05) are left blank.

**Supplementary Figure S2.** PCA scree plot: Explained variance per component (PC1 ≈ 53.6 %, PC2 ≈ 24.5 %; cumulative ≈ 78.1 %).


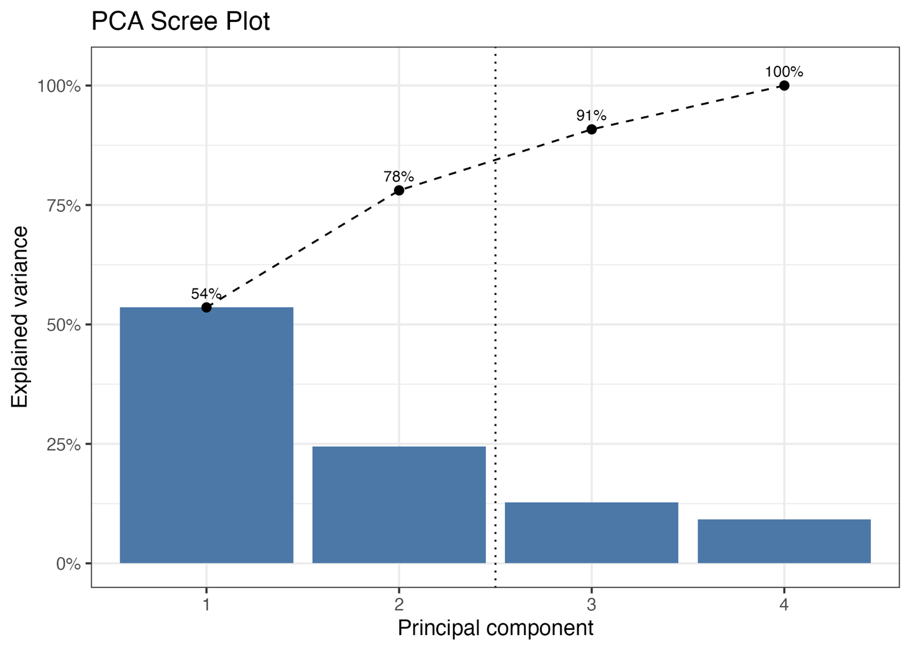


**Supplementary Figure S3**: Bootstrap confidence intervals for index-wise group effects.
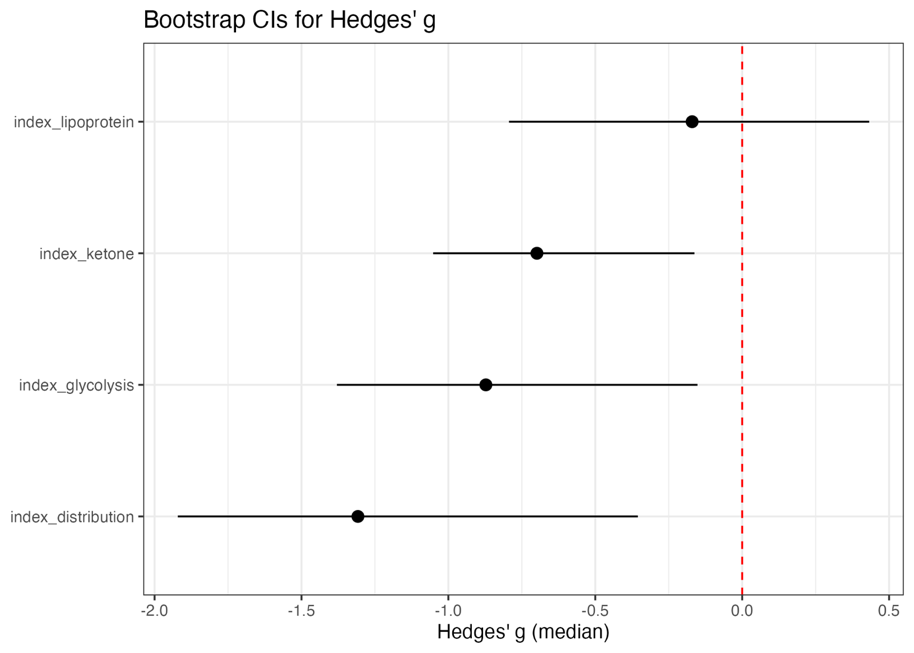

Supplement: Supplementary file 1 [file DataSheet1.docx]
